# Resiliency, the Lived Experience of Patients Undergoing Hematopoietic Stem Cell Transplantation

**Published:** 2019-10-01

**Authors:** Ali Karimi Rozveh, Alireza Nikbakht Nasrabadi, Shahrzad Ghiyasvandian, Leila Sayadi, Mohammad Vaezi, Reza Nabi Amjad

**Affiliations:** 1 School of Nursing and Midwifery, Tehran University of Medical Sciences, Tehran, Iran; 2 Hematology- Oncology and Stem Cell Transplantation Research Center, Tehran University of Medical Sciences, Tehran, Iran; 3 Non-communicable Diseases Research Center, Alborz University of Medical Sciences, Karaj, Iran

**Keywords:** Lived experience, Hematopoetic stem cells, Resiliency, Phenomenology

## Abstract

**Background:** Hematopoetic stem cell transplantation is considered as a standard treatment for cancer patients to stay hopeful toward treatment outcome. However, these patients experience many complications which might affect different aspects of their life. The aim of this study was to investigate the lived experience of patients after hematopoetic stem cell transplantation and introduce supportive care strategies.

**Materials and Methods:** In this study, Van Manen’s Hermeneutic phenomenological approach was used. Eleven patients (7 males and 4 females) were chosen by targeted sampling from visitors of Shariati Hospital’s outpatient clinic. Semi-structured interviews were conducted and the final data were analyzed by MAXQDA 10 software.

**Results:** Data analysis revealed that the main theme was resiliency with two sub-themes of “not surrendering to disease” and “feeling closer to God”.

**Conclusion:** Participants declared that transplantation was like a second chance for life and considered this opportunity as a gift from God to overcome their disease. According to our findings, spirituality aids can help patients control the disturbances following HSCT and health professionals can use constructive strategies to support patients with spiritual needs.

## Introduction

 Hematopoetic stem cell transplantation (HSCT) is the most important and casual transplantation method worldwide. It has been considered as a life giving and last resort procedure for many diseases, especially diseases with high mortality, in past decades^   1 ^. Since the prevalence of diseases which need transplantation are high in Iran ^2^^,^^3^ , this method caught the attention of health policy makers and the first transplantation center was launched in 1991, and their amount and the amount of transplantation procedures have been increasing ever since^   4 ^.

Although the main goal of hematopoetic stem cell transplantation is returning the patients to normal life, but this transplantation is very time-consuming and costly and also the patients might suffer from many physical and psychological complications ^5^^,^^6^ . Initial preparations before HSCT procedure, long duration of post-operative cares and follow ups (months to years after the transplant) and purchasing essential drugs and equipment, might lead to financial problems for both patients and their families ^5^^,^^7^^-^^9^ . Combination of these problems could create a unique experience for patients that underwent HSCT, and this requires more investigation and inspection. It looks like that patients who experience the most difficult physical, psychological, social and financial problems are living in a state of limbo between life and death^   10 ^.

Nurses, which are considered as the primary members of the treatment team, spend more time with patients and have a substantial role in a successful transplantation and optimum supportive cares. Their presence could also make the patients feel that they are being looked after  ^11^^-^^13^ . This task of patient support is best done when the supportive cares are based on conducted investigations and the real experiences and problems of the patients.

## MATERIALS AND METHODS

 In this study, the lived experience of patients after transplant was investigated by Van Manen’s hermeneutic phenomenological approach. This approach is consisted of six steps or main activities which do not necessarily precede each other, but are activities which the researcher must address and accomplish^   14 ^. The first two steps of Van Manen’s approach include establishing the main question of the research, selecting participants and data gathering and the next four steps are associated with data analysis. However, Van Manen mentions that these steps are not separate and all play a role in the practical phenomenological approach^15^^, ^^16^.

To acquire a rich data, we used targeted sampling with maximum diversity. Eleven patients (7 males and 4 females) with average age of 35.9 attending the outpatient clinic of Shariati Hospital were selected for this study. All of these patients were literate and had appropriate physical and psychological health, all of them were above 18 years of age and were able to communicate properly. They had diverse HSCT period duration, from 5 months to 13 months. In order to encourage the patients to participate in the study, the researcher started with questions like “tell us the story of your life after the hematopoetic stem cell transplantation”. For collecting additional information, the researcher asked questions like “could you give us another example?” or “could you please explain it more?” to clarify their answers. Given the principles of qualitative studies, patients from both sexes were included in the study and interviewed. Location, date and duration of each interview were set after consulting with each patient. Each interview session was between 35 to 100 minutes long. All of the interviews were recorded after getting consent from the patients. The interviews were transcripted within 48 hours and were analyzed by Max QDA 10 software. Sampling process was ended when no new theme was found in performed analysis.

In the first step, after contemplation and clarifying the basic assumptions, we focused on the meaning of life after stem cell transplantation. In the second step, the patients experience was investigated by collecting data from interviews and related literature. In the third step, interviews were transcripted by the investigator and they were double checked by listening to recorded interviews to prevent transfer of false data.

In the fourth step, by reflecting on patients’ statements, the investigator demonstrated rich and precise description and interpretation of the patients’ experience. In the fifth step, by considering main questions and supervision of research team on patients’ performance, it was attempted to establish a strong connection with the patients’ experience. During the sixth step, research context is balanced by considering both the partial segments and completed portion.

In order to ensure the value of qualitative data and validate the reliability of the results, four criteria of Lincoln and Guba were used. These four criteria are 1) Credibility, 2) dependability, 3) Conformability and 4) Transferability.

## Results

 The main finding of this study was the answer to the question “What is life after hematopoetic stem cell transplantation?” The relationship between humans and their God has the potential to provide people with a strong spiritual energy which in turn can give them hope, increase their will for life and cultivate their potential abilities: abilities which help them to overcome adversities and achieve great goals. This was the experience that the patients mentioned, which indicates the power of their spiritual beliefs. “Resiliency” was the main emergent theme based on the participants’ statements, which was consisted of two sub-themes, “not surrendering to disease” and “feeling closer to God”.

The emergent main theme and the two sub-themes are presented in Table 1.

**Table 1:** Emergent main theme and sub-themes

**Main theme**	**Sub-themes**
Resiliency	Not surrendering to disease
	Feeling closer to God

**Not surrendering to disease**

Patients increase their ability to survive and overcome difficulties by staying resilient, and consider themselves to be a person with higher capabilities after the transplant. One of the participants commented:

“I changed my profile picture to a picture, saying life is hard, but I am harder; like a tree in a storm. The tree might get damaged or even lose some branches, but it will not go down easily. I also decided not to give up so easily” (participant number 4)

Resistance in the face of disease and having the mentality of a fighter was another topic mentioned by the patients.

“A poet once said that you must live until the flowers are living on, and I said to myself if I am fighting a disease like cancer and there is death at the end of it, then I will try and fight it until I can.” (Participant number 1)

Another participant said: 

“If you don’t have a strong soul, failure may be at the door. Now I feel that I was strong enough to endure to the end.” (Participant number 4)

Another participant pointed out the difficulties of transplantation procedure and said that his view on problems has changed:

“My problems have made me stronger. I believe that difficulties make people more resilient and strong and you are not able to be strong if you don’t face difficult situations. Transplantation was very hard, but I believe that difficult times will pass as well.” (Participant number 2)

Another participant commented about their tolerance:

“If you truly want to stay alive, you must will it. I mean that you must get rid of any doubt or disbelief and think about positive outcomes. You should not think about the disease and where it might lead you to, but instead you must fight for your life and fight anything that stands in your way. You should not be afraid or take it too hard on yourself.” (Participant number 6)

Another participant pointed out the power of positive thinking and believed that the ailments are God’s will:

“You must understand that we do not control everything, every person has his/her own fate and destination. Mine was this way. But you must take care of yourself and do not aggrandize your problems; everyone has problems. You should not use negative thoughts. I believe that everything is in God’s hands.”(Participant number 8)

**Feeling closer to God**

In this study, participants declared that they feel closer to God and felt the presence of God in all of the treatment stages. They adhered to their religious duties during the treatment and tried to act according to God’s rules. One of the participants commented about the presence of God in her life:

“I know God better now. God has watched over me and answered all of my prayers. I believe that God wants the best for all of his children, and we are not aware of this.” (Participant number 2)

Another participant believed that the disease has brought him closer to God:

“I really felt the power of God. I thank God, he has returned me to himself again. I somehow like this disease, because it has brought me closer to God. God showed me that he has not forgotten me.” (Participant number 11)

Participants felt closer to God when they prayed to God and partook in their religious activities. A participant said:

“I feel that my prayers have become more pure and honest. I say my prayers at the right time and I enjoy them very much. I try to do my religious duties properly, read holy Quran, have proper hijab and talk to my God regularly. I thank God because he has chosen me and tested me. I hope that I am a good servant for God. I feel that this disease has brought me closer to God.” (Participant number 2)

A participant thought of the disease as a gift from God and said:

“This disease made me to think more about myself and the things that I have done and it also helped me to know myself better. I believe that this was kind of a test from God, and now I know God better than before. Prays be upon God.” (Participant number 6)

Another participant believed that the disease is God’s will and said:

“At first, I thought to myself that why this has happened to me, but then I realized there must be a reason, there must be a reason for my illness. Now I believe that everything is in God’s hands and I should not doubt God. I have faith in God and I believe that whatever God wants for me, it must be best for me. Well, it’s my fate.” (Participant number 9)

Participants try to adhere to their religious rituals and get blessings from God. They focus on their spiritual activities more. A participant mentioned:

“I read the holy Quran to feel more peaceful, I also say my prayers. I don’t do all of my religious duties, but I do them much more than before. I didn’t used to be a religious person, but now when I talk to God, I feel more at ease and calm.” (Participant number 10)

Many of the participants said that this experience increased their belief in God. For instance, one of them said:

“Now my belief in God has become stronger. I’d like to do more benevolent activities and I don’t like to upset other people. I decided to walk to holy shrine in Karbala after my vaccination, and I will do that!” (Participant number 5)

One of the participants saw the disease as an opportunity for purification and said:

“This is a unique opportunity for purification and I believe that I must not think negative thoughts. I ask for help from God and our prophet, and this way I will never have to stand alone.” (Participant number 10)

## Discussion

 The resiliency theme emerged from participants’ experience, with two sub-themes of “not surrendering to disease” and “feeling closer to God”. Resiliency is the capacity to resist in the face of tensions. Resiliency increases a person’s capacity to adapt to rough situations and overcome dangers and adversities. People even can rebuild their life after devastating accidents. People who go through HSCT experience a great deal of stress when they get diagnosed. Resiliency is one of the approaches which people use to confront difficult situations and adapt after the transplant procedure. Studies have shown that there is a strong link between resiliency and health and life quality of patients and the socio-psychological consequences of surgery after the transplant^     17 ^.

The sub-theme of “not surrendering to disease” emerged based on the participants’ statements. The vulnerability of these patients did not make them to feel defeated in advance. They automatically made their best effort to encounter their severe situation. Each of them, based on their resources, chose a suitable approach to gain strength. 

In a study by Gilfillan (2011), the sub-theme of “Adaptation and managing the impact” emerged from the experiences of participants in facing their disease. Participants showed significant ability in using different skills to confront their disease and regain their health. Their statements indicated their strong will to overcome difficult situations which they were facing. The “fighter” mentality had a strong impact in the patients’ well-being after the transplant^   18 ^. In another study by smith (2017), the theme of countering vulnerability emerged which is similar to the theme in the present study. The participants were actively looking for ways to decrease their vulnerability. Some of the participants explained that they have changed their point of view and perceive this incident as a fighting opportunity, and they must use this to encounter vulnerability ^   19 ^. The main theme in a study by Coolbrandt et al. (2010), was writing positive stories. In this study, participants used positive thinking to stay optimistic and they tried not to focus on negative outcomes^   20 ^. In another study, the theme of “priorities and release control” emerged from participants' statements. Some of the participants believed that the transplantation procedure has affected their worldview. They mentioned that prior to transplant, they had a great deal of anxiety about the future, their families and their job, but following transplantation they figured out that they are not responsible for their disease and they must live in the present moment. The meaning of life had changed for these patients and they tried to change their priorities in life and not to take their life so seriously. They believed that the psychological impact of transplantation made them able to solve their problems by changing their thoughts^21^.

In our study, participants had accepted their new situation and saw it as God’s will. They tried to adapt to their disease and current situation by staying optimistic and having positive mentality. The main reason for this reaction was to shift from a dangerous and harmful place in their life to a more peaceful place to increase their chance of success. Our findings indicate that the HSCT patients moved to a new life after the transplant which is considerably different from their life before the transplant. They saw the radical changes in their spiritual beliefs and their faith as a result of becoming closer to God. They perceived this spiritual purification as a result of their disease and the presence of God in every aspect of their life. The sub-theme of feeling closer to God was the result of these statements made by study participants. They mentioned that after HSCT they adhered strictly to their religious duties and tried to perform other religious rituals such as night prayers as well. In another study, the main theme was using religious/spiritual resources which were similar to our findings. Many of the recipients emphasized the importance of religious rituals in connecting with God. They mentioned that activities such as praying and reciting the holy Quran helped them to overcome difficulties of their situation. Activities like praying and reciting sacred texts is a common adaptation mechanism which increase hope for many people. Adhering to religious duties is a great source of power for patients and it has the potential to accelerate their recovery ^   22 ^. People increase their spiritual activities when facing devastating diseases like cancer. These activities reduce stress and anxiety and increase sense of peace and hope in patients^23^. 

Many of the survivors believe that they have been chosen by God, that God watches over them and their situation is the result of God’s will. They believe that both the disease and the cure is in God’s hands and anything that might happen in their life is also in God’s hands, and this belief brings peace to their mind and heart. In the study by Alaloul et al. (2016), the main theme of “sickness viewed in light of belief in God” emerged and the participants mentioned that their faith in God keeps them safe and protects them in every situation and that their faith makes it easier for them to accept their bitter experience and overcome it. This compromise was the result of four approaches: faith in god is the best cure, having faith in destiny, not viewing disease as misery and being patient. They believed that whoever stays patient will be rewarded in this life and the next life^   22 ^.

Some of the participants considered the life after HSCT as an opportunity for purification and as a chance for contemplation and reconnecting with their God. In a study by Alnasser et al. (2018), the sub-theme of "self-purification” emerged and transplant recipients said that they need the spiritual connection with their god after the transplant and whenever they feel desperate and sad, they recite the holy Quran and this gives them sense of peace and changes their mental attitude. Participants believed that the isolation period is an opportunity to purify their soul. From a religious point of view, the considered the medical treatment only as a means, and believed that the real treatment comes from God, and for this reason they had increased their spiritual connection with God^       21 ^.

According to our observations in the present study, participants believed that they had positive changes after HSCT, and their spiritual life has flourished ever since. This change of view made them focus on the positive sides of their surgery instead of the negative and harmful consequences of their transplant. Therefore, they acquired a better and deeper understanding of their lives and also enhanced their spiritual life. Their experience made them more grateful in their life.

## CONCLUSION

 One of the most important abilities in struggling with cancer is Resilience. It gives people the power to confront their challenges and overcome adversities. It also helps people to resist against the tensions. Our study gave us accurate information about the experience of HSCT patients after their transplant. Participants of our study said that the disease has affected every aspect of their life. They had accepted their disease and felt the need to rebuild their life. They believed that by relying on their religious beliefs, they will gain the power to deal with their dire situation. Spiritual consultation should be among the basic services which health professionals provide for these patients. Presence of religious counselors could be a starting point for incorporating spiritual health into health system.

## STUDY LIMITATIONS

Although visitors of Shariati Hospital are from all around the country, but the samples of the present study do not represent every population and culture in Iran. Like any other qualitative study, there were some limitations regarding generalization of the results.
